# Sarcomatoid Carcinoma in a Resected Hepatic Metastasis of Esophageal Squamous Cell Carcinoma after Chemotherapy

**DOI:** 10.70352/scrj.cr.25-0257

**Published:** 2025-09-17

**Authors:** Kotaro Kimura, Yoshihiro Kinoshita, Naoya Okada, Takumi Yamabuki, Saki Kawaguchi, Chiho Goto, Ryouto Koyama, Yusuke Asai, Zen Naito, Hiroyuki Yamamoto, Tomohiro Suzuki, Yusuke Tsunetoshi, Noriaki Kyogoku, Hirokatsu Katagiri, Kentaro Kato, Minoru Takada, Yoshiyasu Ambo, Yuko Omori

**Affiliations:** 1 Department of Surgery, Teine Keijinkai Hospital, Sapporo, Hokkaido, Japan; 2 Department of Pathology, Teine Keijinkai Hospital, Sapporo, Hokkaido, Japan

**Keywords:** sarcomatoid carcinoma, esophageal squamous cell carcinoma, epithelial-mesenchymal transition, neuroendocrine differentiation

## Abstract

**INTRODUCTION:**

Sarcomatoid carcinoma is a rare histological variant of carcinoma characterized by a mesenchymal-like morphology, often arising through the epithelial-mesenchymal transition. Although sarcomatoid carcinoma is occasionally observed in primary esophageal carcinosarcoma, its diagnosis at metastatic sites is rare.

**CASE PRESENTATION:**

A man in his 60s was diagnosed with esophageal squamous cell carcinoma with synchronous liver and cutaneous metastases. A cutaneous nodule in the abdomen was surgically resected and pathologically confirmed to be metastatic squamous cell carcinoma. Owing to the unresectable nature of the tumor, the patient underwent 10 cycles of combined pembrolizumab and fluorouracil + cisplatin therapy, followed by 20 cycles of S-1 monotherapy owing to immune-related adverse events. This treatment resulted in a complete clinical response of the primary esophageal tumor and a significant reduction in most liver metastases. However, one liver metastasis in the left lateral segment exhibited progressive disease, requiring surgical resection. Pathological examination of the hepatic lesion revealed a well-demarcated lobulated tumor with necrosis and hemorrhage. Microscopically, the tumor comprised polygonal-to-short, spindle-shaped atypical cells with pleomorphism, frequent mitotic figures, and sinusoidal infiltration. Immunohistochemically, the hepatic lesion exhibited diffuse vimentin positivity, partial Cam5.2, and scattered INSM-1 expression, suggesting a mesenchymal transition of the carcinoma with partial neuroendocrine differentiation. CK AE1/AE3, CK5/6, and p40 were negative. *p53* and BRM/SMARCA2 showed a complete loss of expression. A retrospective analysis of esophageal and cutaneous lesions revealed a progressive loss of BRM/SMARCA2 expression across different metastatic sites, supporting the hypothesis of epithelial-mesenchymal transition-driven transformation during hepatic metastasis. Despite surgical intervention, multiple hepatic recurrences were detected within 2 months postoperatively, highlighting the aggressive nature of sarcomatoid carcinoma and the limitations of surgery alone in controlling this disease.

**CONCLUSIONS:**

This case highlights the rare phenomenon of histological transformation of squamous cell carcinoma to sarcomatoid carcinoma within a metastatic site, emphasizing the importance of surgical resection for both diagnosis and treatment. The loss of BRM/SMARCA2 may have contributed to the epithelial-mesenchymal transition-driven transformation and, together with neuroendocrine differentiation, may have played a role in the tumor’s aggressiveness. Histological reassessment of chemotherapy-resistant lesions is crucial for elucidating tumor evolution and optimizing future therapeutic strategies.

## Abbreviations

CPS
: combined positive score
EMT
: epithelial-mesenchymal transition
FP
: fluorouracil + cisplatin
NED
: neuroendocrine differentiation
SC
: sarcomatoid carcinoma
SCC
: squamous cell carcinoma

## INTRODUCTION

SC is an undifferentiated tumor characterized by epithelial-derived cancer cells and sarcomatous components with a mesenchymal-like morphology, thought to develop through EMT.^1,2)^ Unlike carcinosarcoma, which consists of distinct epithelial and mesenchymal components, SC retains epithelial lineage markers and exhibits sarcomatous morphology.^1,2)^ Although SC has been reported in approximately 2% of primary esophageal tumors, its occurrence at metastatic sites is extremely rare.^3)^ Here, we present a case of SC diagnosed as a chemotherapy-resistant hepatic metastasis from esophageal SCC.

## CASE PRESENTATION

**Fig. 1:**
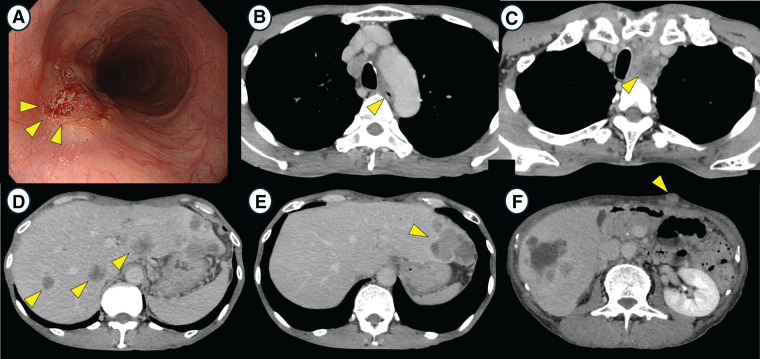
Imaging findings at the time of initial diagnosis (**A**) Upper gastrointestinal endoscopy showing a Borrmann type 3 tumor in the middle thoracic esophagus (arrows). Biopsy confirmed moderately differentiated SCC. (**B**) Contrast-enhanced CT (CECT) indicating the location of the esophageal tumor (arrow). (**C**) An enlarged 106recL lymph node (arrow), suggesting lymph node metastasis. (**D**) Multiple liver metastases (arrows). (**E**) A metastatic lesion in the left lateral segment of the liver (arrow). (**F**) A subcutaneous nodule in the abdomen, which was pathologically diagnosed as a cutaneous metastasis of poorly differentiated SCC (arrow). SCC, squamous cell carcinoma

A man in his 60s with pharyngeal discomfort was referred to our hospital with suspected esophageal cancer. Upper gastrointestinal endoscopy revealed a circumferential one-third type 3 lesion in the middle thoracic esophagus (**Fig. 1A**), and biopsy confirmed moderately differentiated SCC. Imaging diagnosis revealed lymphadenopathy around the left recurrent laryngeal nerve lymph node (station 106recL, according to the Japanese Classification of Esophageal Cancer), tracheal invasion, multiple liver metastases (**Fig. 1B**–**1E**), and synchronous hypopharyngeal cancer. In addition, a 2-cm subcutaneous nodule in the abdomen was resected and pathologically diagnosed as a poorly differentiated SCC, indicating cutaneous metastasis from the esophageal cancer (**Fig. 1F**). The esophageal cancer was staged as cT4bN3M1 (Stage IVB) according to the UICC TNM classification, 8th edition. (In the Japanese classification, the lymph node involvement is considered as N4 due to its anatomic location.) Immunohistochemical analysis of the primary esophageal tumor revealed a PD-L1 expression level with a CPS of 1–10. PD-L1 expression in the metastatic lesions was not assessed.

**Fig. 2:**
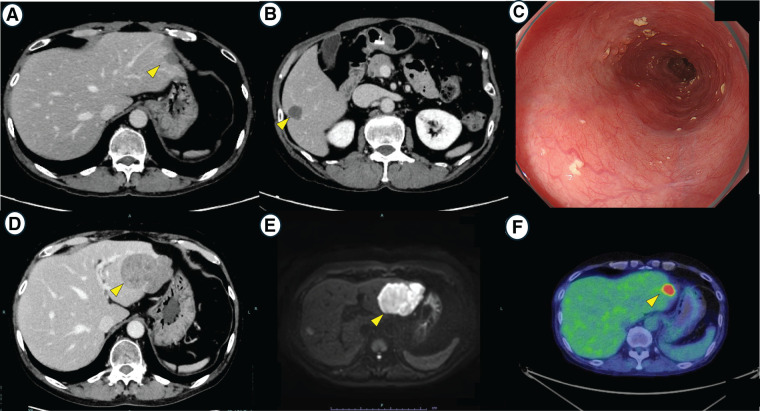
Imaging findings after chemotherapy (**A**, **B**) CT imaging after 10 cycles of pembrolizumab plus FP therapy. Contrast-enhanced CT scans demonstrate a marked reduction in the size of multiple hepatic metastases following combined pembrolizumab and 5-FU/cisplatin therapy (arrow). (**C**–**E**) Imaging after completion of 20 cycles of S-1 monotherapy. (**C**) Upper gastrointestinal endoscopy demonstrating complete regression of the esophageal tumor, indicating a clinical complete response. (**D**) CECT showing that most liver metastases have regressed, except for a residual tumor in the left lateral segment of the liver (arrow). (**E**) Diffusion-weighted imaging on MRI showing high signal intensity in the residual hepatic lesion (arrow). (**F**) PET-CT performed approximately 4 months earlier (**C**–**E**) revealing intense 18F-fluorodeoxyglucose uptake in the left lateral hepatic lesion (standardized uptake value max 11), consistent with persistent tumor activity (arrow). At the time of imaging, the lesion was small, and the uptake was observed throughout the tumor. FP, fluorouracil + cisplatin; CECT, Contrast-enhanced CT; PET-CT, positron emission tomography-CT

**Fig. 3:**
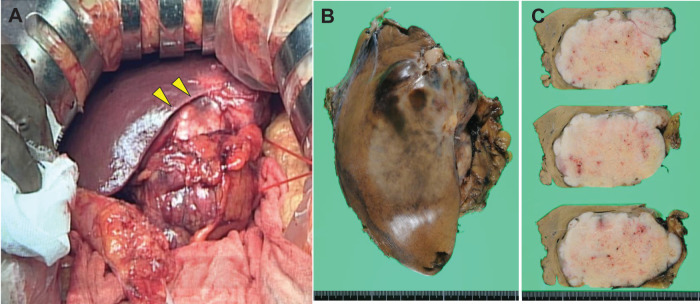
Gross pathology of the hepatic metastasis (**A**) Intraoperative finding of the tumor in the left lateral segment (arrows). (**B**) Fixed hepatectomy specimen showing a solid tumor protruding from the liver surface measuring 105 × 85 × 50 mm in size. (**C**) Cross-sectional inspection revealed a well-demarcated, lobulated, firm nodular mass with a yellow-white hue, showing necrosis and hemorrhage. Peripheral daughter nodules were observed.

Due to the unresectable nature of the tumor, a combination of pembrolizumab and FP therapy was initiated. After 10 cycles of pembrolizumab plus FP therapy (**Fig. 2A** and **2B**), the patient developed grade 3 pruritus, considered as a cutaneous immune-related adverse event. Despite topical and oral treatments at maximal doses, symptoms persisted. Although a trial of prednisolone (10–15 mg) with continued pembrolizumab was proposed, the pruritus did not improve, and pembrolizumab was discontinued. The patient was subsequently switched to S-1 monotherapy at 80 mg/m^2^, which was administered for 20 cycles. Endoscopic evaluation revealed a complete clinical response of the primary esophageal lesion (**Fig. 2C**), with significant shrinkage of most lymph nodes and liver metastases. However, a solitary liver metastasis in the left lateral segment exhibited progressive growth and was considered to warrant surgical intervention (**Fig. 2D**–**2F**); therefore, a left lateral hepatic segmentectomy was performed. Intraoperative ultrasonography was used to confirm that the hepatic lesion was solitary and no additional metastases were present. The procedure lasted 207 min, blood loss was minimal, and the patient was discharged on POD 9 without complications (**Fig. 3A**).

The hepatic lesion was a well-demarcated, lobulated, firm, nodular mass with a yellow-white hue measuring 105 × 85 × 50 mm (**Fig. 3B** and **3C**). The tumor exhibited necrosis and hemorrhage with peripheral daughter nodules.

**Fig. 4:**
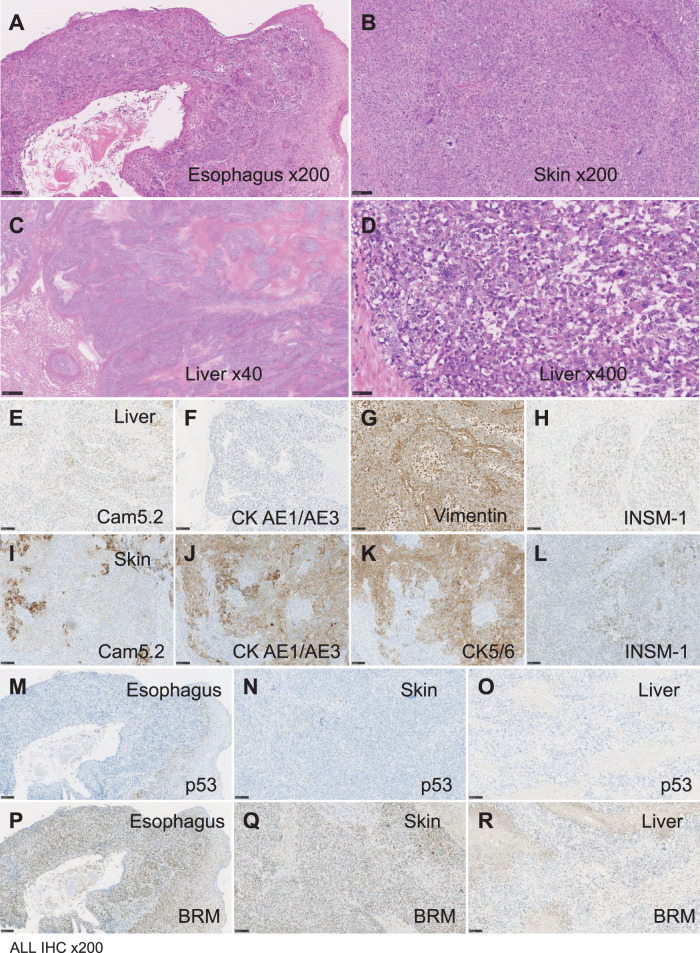
Histological and immunohistochemical analysis of the primary esophageal cancer, cutaneous metastasis, and hepatic metastasis (**A**–**D**) Hematoxylin and eosin staining of the esophageal tumor (**A**), cutaneous metastasis (**B**), and hepatic metastasis (**C**, **D**). The hepatic lesion (**D**) revealed polygonal-to-short, spindle-shaped atypical cells with enlarged nuclei and prominent nucleoli. Tumor cells proliferated in a medullary pattern with extensive coagulative necrosis. Multinucleated and giant cells were present, along with frequent mitotic figures. The cells showed no keratinization or intercellular bridges, and they infiltrated the sinusoids. Immunohistochemical staining for Cam5.2 (**E**), CK AE1/AE3 (**F**), vimentin (**G**), and INSM-1 (**H**) in hepatic metastasis and Cam5.2 (**I**), CK AE1/AE3 (**J**), CK5/6 (**K**), and INSM-1 (**L**) in cutaneous metastasis. Complete loss of *p53* expression was observed in the esophageal tumor (**M**), cutaneous metastasis (**N**), and hepatic metastasis (**O**). BRM/SMARCA2 staining was retained in the esophageal tumor (**P**), partially attenuated in cutaneous metastasis (**Q**), and completely lost in the hepatic lesion (**R**). Images were captured at a magnification of x200 for **A**, **B**, and **E**–**R**, x40 for **C**, and x400 for** D**. CK, cytokeratin

Histologically, the biopsy of the esophageal tumor at the initial examination revealed SCC (**Fig. 4A**). The skin nodules were diagnosed as poorly differentiated SCC (**Fig. 4B**). The liver lesion consisted of polygonal to short spindle-shaped atypical cells with enlarged nuclei and prominent nucleoli, proliferating in a medullary pattern with extensive coagulative necrosis (**Fig. 4C**). The tumor exhibited pleomorphisms, including multinucleated and giant cells with frequent mitotic figures (**Fig. 4D**). No evidence of keratinization or intercellular bridges was observed. In addition, no distinct mesenchymal ectopic tissue, such as bone or cartilage, was observed. Tumor cells infiltrated the sinusoids.

Immunohistochemically, the hepatic lesion exhibited diffuse positivity for vimentin, partial positivity for Cam5.2 and D2-40, and scattered positivity for INSM-1 (**Fig. 4E**, **4G**, and **4H**). CK AE1/AE3, CK 34βE12, CK5/6, p40, and hCG were negative (**Fig. 4F**). *p53* and BRM showed complete loss of expression, whereas Brahma-related gene 1 and integrase interactor 1 retained their expression (**Fig. 4O** and **4R**). These findings led to the diagnosis of sarcomatoid carcinoma with partial neuroendocrine differentiation (NED).

To assess the histological evolution of the tumor, primary esophageal tumors and cutaneous metastases were retrospectively analyzed. Esophageal biopsy revealed a moderately differentiated SCC with small cancer pearl formation and individual cell keratinization (**Fig. 4A**). Immunohistochemically, loss of *p53* expression and partial attenuation of BRM were observed (**Fig. 4M** and **4P**). In the cutaneous metastasis, poorly differentiated SCC with minimal keratinization was noted, with positive staining for CK AE1/AE3, Cam5.2, and CK5/6 (**Fig. 4B**, and **4I**–**4L**). *p53* expression was absent, whereas BRM exhibited partial attenuation to loss of expression (**Fig. 4N** and **4Q**). The INSM-1 cells were sparsely positive (**Fig. 4L**).

Given the shared *p53* loss across all lesions and the progressive attenuation and loss of BRM/SMARCA2 expression, it was concluded that the hepatic tumor originated from an esophageal SCC and underwent a sarcomatoid transformation at the metastatic site. Inactivation of BRM/SMARCA2 might have contributed to decreased differentiation and sarcomatoid phenotypic transition.

**Fig. 5:**
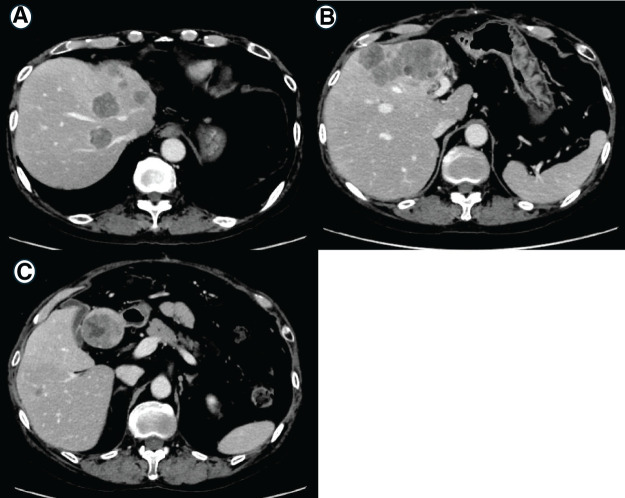
CT imaging obtained 2 months after surgery (**A**–**C**) Contrast-enhanced CT showing multiple new hepatic metastases indicating early recurrence following hepatic resection. These findings prompted the initiation of systemic chemotherapy.

Postoperatively, the patient was monitored without adjuvant therapy; however, multiple hepatic recurrences developed 2 months later, requiring the resumption of chemotherapy (**Fig. 5**). Considering the sarcomatoid and neuroendocrine features of the tumor, a regimen for extensive-stage small cell lung cancer—durvalumab (Imfinzi), etoposide, and cisplatin—was selected. Neuron-specific enolase (NSE) was used as a tumor marker. Although a temporary decrease in NSE was observed, the patient developed drug-induced pneumonitis shortly after treatment initiation, which was attributed to durvalumab and led to its discontinuation. Tumor progression was noted after 2 months, and the patient subsequently opted for best supportive care. He died 6 months after surgery.

## DISCUSSION

Here, we report a rare case of SC identified as a hepatic metastasis of esophageal SCC. Following chemotherapy for SCC of the esophagus and synchronous liver metastases, a solitary hepatic lesion exhibiting progressive growth was resected and subsequently diagnosed as SC.

SC, first described by Virchow in 1864, is a poorly differentiated tumor with sarcomatous morphology.^3)^ Unlike carcinosarcomas, which consist of histologically distinct epithelial and mesenchymal components, SC exhibits a more gradual transition between the carcinoma and sarcomatous components. This supports the hypothesis that sarcomatous transformation in SC arises from carcinoma via EMT rather than from a separate mesenchymal lineage.^1,2)^ Histologically, SC often exhibits poorly differentiated cancer components, which sometimes retain their nest-like structures. Immunohistochemically, SC typically expresses cytokeratin (AE1/AE3), whereas undifferentiated sarcomatous components express vimentin.^3)^

In this case, the esophageal tumor presented as a type 3 lesion and was diagnosed as SCC. By contrast, hepatic metastasis results in the loss of BRM/SMARCA2 expression, a key component of the SWI/SNF chromatin remodeling complex essential for regulating gene expression and maintaining epithelial cell identity.^4)^ This deficiency likely drives the phenotypic transformation of SCC to SC by promoting EMT.^5)^ It also facilitates tumor plasticity, invasiveness, and therapy resistance by downregulating adhesion molecules, such as E-cadherin.^5,6)^ Additionally, the shared loss of *p53* expression across the primary lesion, cutaneous metastasis, and hepatic lesions suggests a clonal relationship. *p53* plays a critical role in maintaining genomic stability and suppressing tumor progression.^7)^ Its deficiency disrupts the transforming growth factor/Smad signaling pathway, a key regulator of EMT, thereby promoting mesenchymal-like characteristics and tumor plasticity.^8,9)^ The combined loss of BRM/SMARCA2 and *p53* likely synergizes, driving the phenotypic shift from SCC to SC and contributing to treatment resistance.^8,10)^ Furthermore, *p53* mutations are associated with impaired DNA repair mechanisms and increased susceptibility to selective pressure, suggesting that chemotherapy may exacerbate this transformation.^7,11)^ PD-L1 expression has been reported to be associated with EMT in esophageal SCC.^12)^ In the present case, the primary tumor exhibited a PD-L1 CPS of 1–10, suggesting a low to intermediate level of expression. However, PD-L1 status in the metastatic lesions, particularly the hepatic metastasis, was not evaluated, which is a limitation in assessing the immunologic and phenotypic changes during disease progression.

The decision to proceed with surgical resection was based on the existing evidence suggesting that local control improves outcomes in patients with oligometastatic disease.^13)^ Hepatic resection has been associated with prolonged survival in well-selected patients, particularly those with controlled systemic disease and limited liver involvement.^13)^ In esophageal cancer, patients who achieve a significant response to systemic therapy may become candidates for conversion therapy, in which initially unresectable tumors are re-evaluated for surgical intervention. However, histological transformation in chemotherapy-resistant lesions complicates the selection criteria for surgery as it may alter tumor biology and lead to unexpected outcomes. In this case, the progressive growth of the hepatic lesion, despite systemic therapy, justified surgical intervention. Although a CT-guided liver biopsy was technically feasible, it was not considered preoperatively. The patient initially had multiple liver metastases, but all lesions except one regressed with systemic therapy, leaving a single, well-demarcated lesion in the left lateral segment. Given the resectability of the tumor and the potential prognostic benefit of local control in the context of oligometastatic disease, surgical resection was prioritized over biopsy. Therefore, resection was pursued without prior biopsy. However, given the early postoperative recurrence, a biopsy, rather than surgical resection, might have been a more appropriate approach. This highlights the challenge of distinguishing true oligometastatic disease from the aggressive residual disease that has undergone a histological transformation. In esophageal cancer, oligometastasis lesions that respond well to systemic therapy tend to have better outcomes following resection.^14,15)^ However, when a metastatic lesion exhibits progressive growth despite systemic therapy, a preoperative histological reassessment should be considered to optimize the treatment strategy. This procedure provides both therapeutic benefits and critical diagnostic insights into histological transformation. Nevertheless, the rapid recurrence suggests that surgical intervention alone is insufficient for long-term disease control, reinforcing the need for a more comprehensive treatment approach for similar cases.

Partial NED, as indicated by INSM-1 positivity, suggests a unique biological behavior. NED is associated with increased tumor aggressiveness, resistance to conventional therapies, and poor prognosis in various malignancies.^16)^ The expression of neuroendocrine markers, such as chromogranin A, has been shown to enhance tumor plasticity and invasiveness by secreting biologically active substances. Furthermore, NED has been identified as an independent prognostic factor for poor outcomes, particularly in advanced-stage tumors.^16)^ In this case, surgical resection temporarily achieved local disease control, leading to the decision to forgo adjuvant chemotherapy and proceed with follow-up monitoring. However, multiple metastatic recurrences were observed just 2 months after surgery, suggesting that NED might have influenced the rapid progression of the disease. Given its potential to alter tumor biology, NED may serve as a biomarker for identifying aggressive subtypes and guiding personalized treatment strategies in patients with SCC. In our case, the hepatic metastatic lesion exhibited partial NED, supported by immunohistochemical positivity for INSM-1. While the exact mechanism underlying the emergence of NED remains unclear, it is plausible that prior treatments, including chemotherapy and immune checkpoint inhibitor therapy, played a role in inducing this phenotypic change. Although there are reports of esophageal SCC transforming into neuroendocrine carcinoma following neoadjuvant immunochemotherapy,^17)^ and studies have described the coexistence or sequential occurrence of EMT and NED in other malignancies, such as prostate and lung cancers,^18)^ to our knowledge, no studies have directly documented simultaneous EMT and NED in esophageal SCC. Nonetheless, these observations support the hypothesis that therapeutic interventions may enhance tumor cell plasticity, potentially leading to distinct phenotypic transformations. Further investigations are warranted to clarify the biological mechanisms underlying such treatment-induced changes and their clinical significance.

This case underscores the importance of histological reassessment of chemotherapy-resistant lesions and demonstrates the use of surgical resection for both diagnosis and treatment planning. Future studies should focus on refining the selection criteria for resection in oligometastatic esophageal cancer cases following systemic therapy, particularly for tumors with suspected histological transformations. The integration of advanced imaging modalities, liquid biopsy techniques, and molecular profiling may help distinguish indolent oligometastases from aggressive, treatment-resistant clones, thereby guiding optimal treatment decisions. Further studies are warranted to clarify the optimal management of such cases, investigate the mechanisms underlying EMT-driven histological transformation, and explore the clinical implications of NED in tumor progression and treatment resistance.

## CONCLUSIONS

This case highlights the rare histological transformation of esophageal SCC into SC at a metastatic site following chemoradiotherapy. The loss of BRM/SMARCA2 and *p53* likely contributed to EMT and the emergence of an aggressive sarcomatoid phenotype. Partial NED may have further contributed to tumor progression, treatment resistance, and early recurrence.

Surgical resection provided critical diagnostic insights; however, it was insufficient for long-term disease control, as evidenced by early recurrence. Preoperative histological reassessment of chemotherapy-resistant lesions is essential for optimizing treatment decisions and improving long-term outcomes. Given the rapid recurrence observed in this case, close postoperative surveillance and consideration of adjuvant therapy may be warranted in similar cases exhibiting sarcomatoid transformation with NED.

## DECLARATIONS

### Availability of data and material

The datasets used or analyzed during the current study are available from the corresponding author upon reasonable request.
